# Anisotropy in Carbon Dioxide Adsorption on Forsterite

**DOI:** 10.3390/ijms252312639

**Published:** 2024-11-25

**Authors:** Yakov Ermolov, Andrey Vasilchenko, Georgy Lazorenko

**Affiliations:** 1 Climate Center, Novosibirsk State University, Pirogov Street, 2, Novosibirsk 630090, Russia; mr.yak.erm@gmail.com (Y.E.); zmandr@mail.ru (A.V.); 2 Technological Faculty, Platov South-Russian State Polytechnic University (NPI), Prosveshcheniya St., 132, Rostov Region, Novocherkassk 346428, Russia; 3 Department of Civil Engineering, Rostov State Transport University, Narodnogo Opolcheniya Sq., Rostov-on-Don 344038, Russia

**Keywords:** forsterite, Mg_2_SiO_4_, binding mechanism, CO_2_, density functional theory

## Abstract

In this study, density functional theory (DFT) method were used to investigate the adsorption behavior and binding mechanism of CO_2_ molecules on six crystallographic surfaces of forsterite (Mg_2_SiO_4_). The influence of surface crystallographic orientation on CO_2_ adsorption efficiency was examined at the atomic level. Results showed stable binding of CO_2_ on all surfaces. The interaction strength decreases in the order: (001) > (101) > (120) > (111) > (010) > (110), with the (001) surface exhibiting the highest binding capacity due to accessible magnesium cations interacting with CO_2_. Detailed electronic property analysis revealed significant charge transfer between CO_2_ oxygen atoms and surface magnesium atoms, driven by hybridization of oxygen 2*p* and magnesium 2*s* orbitals, leading to the formation of ionic and covalent bonds. These interactions stabilize the adsorbed CO_2_ and are accompanied by changes in the electronic structure, such as energy level shifts and modifications in the partial density of states (PDOS). The computational analysis provides a theoretical foundation for understanding CO_2_ binding mechanisms by forsterite. The findings highlight the importance of crystallographic orientation and electronic properties of the mineral surface in adsorption efficiency, contributing to a deeper understanding of CO_2_ interactions with mineral surfaces.

## 1. Introduction

The increasing concentration of carbon dioxide (CO_2_) in the atmosphere is one of the key factors of global climate change, which makes the tasks of its capture and storage highly relevant [1,2,3]. CO_2_ mineralization is an effective method for CO_2_ capture and storage because it provides long-term immobilization of CO_2_ [4]. While the initial binding of CO_2_ is a crucial initial step in mineralization, the formation of stable carbonates like magnesite (MgCO_3_) depends on various environmental conditions and subsequent chemical reactions. Understanding the fundamental binding mechanisms and energetics at the atomic level can provide insights into how these initial interactions may facilitate or hinder the formation of stable carbonate minerals under different conditions.

Olivine (Mg^2^⁺, Fe^2^⁺)_2_SiO_4_, which is widely distributed in basalts, is a promising material for CO_2_ mineralization due to its high content of metal cations and weak metal-oxygen bonds caused by its fully unpolymerized tetrahedral silicate structure [5,6]. Minerals of the olivine group, particularly forsterite (Mg_2_SiO_4_), are considered effective for CO_2_ mineralization due to their low cost, wide availability, and ability to chemically react with CO_2_ [7,8,9]. Forsterite, the magnesium-rich end member of the olivine series, actively participates in CO_2_ mineralization processes, where carbon dioxide chemically reacts with the mineral to form stable carbonates such as magnesite (MgCO_3_), contributing to the long-term storage of CO_2_ [10,11]. 

A critical initial step in the mineralization process is the binding of CO_2_ onto the mineral surface, as it initiates subsequent chemical reactions leading to carbonate formation. Therefore, understanding the binding mechanisms of CO_2_ on forsterite surfaces is essential for optimizing mineral carbonation processes. Previous experimental and theoretical studies have explored interactions of forsterite with various molecules, including water, glycine, ammonia, CO, and CH_4_ [12,13,14,15,16]. However, many of these studies have focused on physical interactions or initial dissolution steps, without thoroughly investigating the detailed mechanisms of CO_2_ adsorption at the atomic level [17,18,19,20,21].

These studies often use simplified models and reactions, making a detailed understanding of the energetics and surface morphology of forsterite at the atomic level extremely important to fully unravel the nature of these processes, as well as other mineral interface-related phenomena [22]. In addition to experimental approaches, theoretical calculations have been used to investigate forsterite mineralization. For example, the adsorption characteristics of water, CO and CO_2_ molecules on the forsterite surface have been studied using molecular dynamics (MD) [23] and density functional theory (DFT) methods [6,24,25,26,27,28]. Kerisit et al. [24] simulated the optimal configurations of adsorbed water on the (010) forsterite surface. Their MD simulation results revealed that when CO_2_ reacts with forsterite, the molecule does not interact directly with the surface but forms H_x_CO_3_^(2−x)−^ complexes that interact with dissolved metal cations in the water film at the CO_2_-forsterite interface. Kerisit et al. further extended their study to five olivine minerals, finding that the ionic nature of alkaline earth olivines results in stronger exothermic adsorption of water and CO_2_ compared to transition metal olivines, with calcioolivine (Ca_2_SiO_4_) showing the strongest interaction [6]. Additionally, theoretical studies using force fields have been conducted for the adsorption of H, H_2_, and H_2_O on the (010) forsterite surface [29,30,31,32,33,34]. However, a comprehensive comparative study of different surfaces is required to fully understand the mechanisms of CO_2_ mineralization on forsterite, as experimental and theoretical studies show that various crystallographic surfaces of forsterite exhibit different reactivities [22,35]. These differences are due to variations in the mineral’s surface energy and electronic structure [36].

In this work, a systematic study of CO_2_ molecule adsorption on six different forsterite surfaces (010), (101), (111), (001), (110), and (120) was carried out using the DFT method. The equilibrium geometries for these forsterite faces were determined. The geometries of adsorption configurations, adsorption energies, electron density distributions, and partial densities of electronic states were analyzed. This study provides a deeper understanding of the CO_2_ binding mechanism on forsterite surfaces and a theoretical basis for explaining changes in the mineral’s physical properties after interaction with CO_2_. Understanding these interaction mechanisms is a critical step toward optimizing CO_2_ mineralization processes. The findings highlight the importance of crystallographic orientation and electronic properties of the mineral surface in binding efficiency, contributing to a deeper understanding of CO_2_ interactions with mineral surfaces. 

## 2. Results and Discussion

### 2.1. Adsorption Conformation

**Figure 1:**
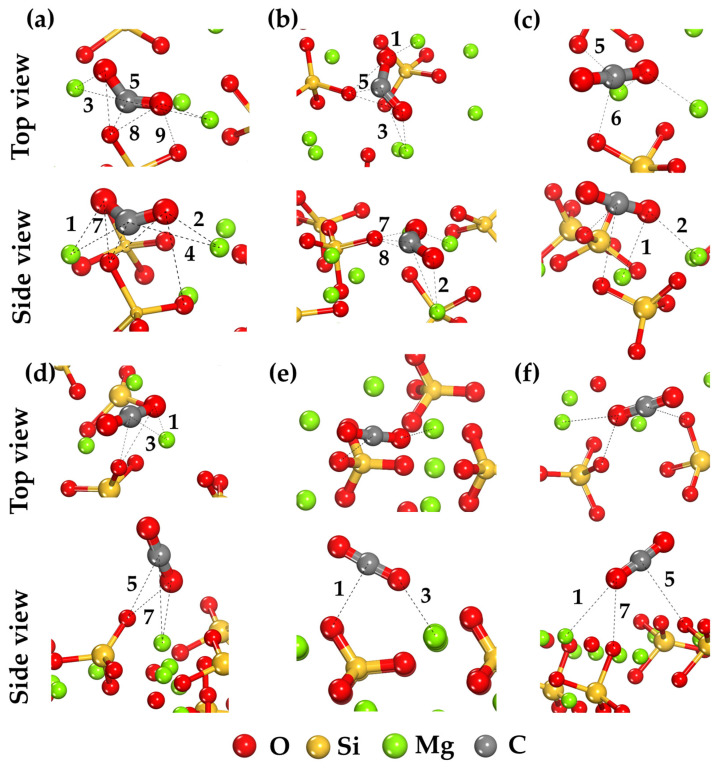
The stable adsorption configurations of CO_2_ molecule on forsterite surfaces: (**a**) (001), (**b**) (101), (**c**) (120), (**d**) (111), (**e**) (010), (**f**) (110). Numbers indicate found bonds (Table 1).

To investigate the adsorption behavior and determine the CO_2_ binding mechanism on different forsterite surfaces, configurations with the lowest energy were selected (Figure 1). States with different orientations of the CO_2_ molecule relative to the mineral surfaces were analyzed. Geometry optimization showed that the CO_2_ orientation has a more significant effect on the interaction energy with forsterite than the specific binding site on its surfaces.

On surfaces with accessible magnesium cations, such as (001) and (101), the most stable configurations are achieved when one or both oxygen atoms of CO_2_ interact with surface Mg atoms. This leads to the formation of a greater number of significant bonds and, consequently, to stronger interaction (Figure 1a,b). For other surfaces where Mg cations are less accessible, low-energy configurations are achieved when CO_2_ interacts predominantly with surface oxygen atoms (Figure 1c–f).

To evaluate the nature of the interaction between CO_2_ and forsterite surfaces, Mulliken bond population analysis was performed, which allows determining the nature of the emerging bonds—covalent or ionic [37]. Non-zero population values suggest the presence of a covalent bond, while values close to zero indicate an ionic or physical nature of the interaction [38].

The nature of bonding interactions varies significantly across different forsterite surfaces, primarily depending on the accessibility of Mg cations and the surface termination. On the (001) and (101) surfaces, where Mg cations are readily accessible, the interaction mechanism involves both ionic and covalent components. The high Mulliken bond population values for Mg–O bonds (0.89–1.28) indicate significant orbital overlap and electron sharing, characteristic of partial covalent bonding. This hybridization of the CO_2_ oxygen 2*p* orbitals with the surface Mg 2*s* orbitals contributes to the formation of strong chemical bonds. The ionic component is evidenced by the substantial charge transfer from CO_2_ to the surface Mg cations, as shown by the Mulliken charge analysis.

In contrast, surfaces with predominantly oxygen termination, such as (110) and (010), exhibit markedly different bonding characteristics. The low Mulliken bond population values (0.01–0.25) suggest minimal orbital overlap and electron sharing. On these surfaces, the interaction is dominated by electrostatic and van der Waals forces, with limited covalent character. The absence of accessible Mg cations restricts the formation of strong chemical bonds, resulting in predominantly physical binding mechanisms.

The largest number of significant bonds is formed on the (001) surface (Figure 1a). The highest bond population is observed for Mg–O bonds, indicating a partially covalent nature of the interaction between surface Mg cations and CO_2_ oxygen atoms. For the additional forming Mg–C, O–C, and O–O bonds, the population decreases (Table 1). This indicates a significant contribution of Mg–O bonds to the binding mechanism of the CO_2_ molecule. The highest bond population values predominate for bonds primarily on the (001), (101), and (120) surfaces. The magnitude of bond population decreases with the reduction in the number of bonds on different surfaces from (001) to (110), indicating a decrease in CO_2_ interaction with the mineral surface (Table 1).

The change in CO_2_ molecule geometry after adsorption on forsterite surfaces is an important indicator of the strength and nature of the interaction between the molecule and the surface. Analysis of the structural parameters of adsorbed configurations shows that the degree of CO_2_ molecule deformation depends on the type of surface where binding occurs, as reflected in Table 2. In the free state, the CO_2_ molecule has a linear structure with C–O bond lengths of 1.180 Å and an O–C–O bond angle close to 180° (179.753°). After adsorption on forsterite surfaces, changes in C–O bond lengths and O–C–O angle are observed, which depend on the surface type and, consequently, on the strength of interaction between CO_2_ and the surface. The increase in C–O bond lengths and decrease in O–C–O angle on these surfaces indicates strong interaction between the CO_2_ molecule and surface Mg cations. This is associated with electron density transfer from the molecule to the surface, leading to the weakening of the C–O bond and deviation of the molecule from its linear configuration (Table 2).

The most significant changes in CO_2_ molecule geometry are observed on the (001) and (101) surfaces, where Mg cations are most accessible for interaction. Data from Table 2 confirm that the most substantial changes in CO_2_ molecule geometry occur on the (001) and (101) surfaces. Here, the C–O bond lengths increase by approximately 0.087–0.090 Å compared to the free molecule, and the O–C–O angle decreases by more than 50°, indicating significant molecular deformation. The increase in C–O bond lengths and decrease in O–C–O angle on these surfaces indicate strong interaction between the CO_2_ molecule and surface Mg cations. This is associated with electron density transfer from the molecule to the surface, leading to C–O bond weakening and deviation from the linear configuration (Table 2).

On the (120), (111), (010), and (110) surfaces, where Mg cations are less accessible or direct interactions with them are absent, changes in CO_2_ molecule geometry are less significant. On these surfaces, changes in C–O bond lengths do not exceed 0.013–0.024 Å, and the O–C–O angle decreases by only 2–4°, indicating minor deformation and weak interaction (Table 2). These minimal changes in CO_2_ molecule geometry indicate weak interaction, predominantly of van der Waals character, with surface oxygen atoms. The absence of significant electron density transfer results in the preservation of the molecule’s linear structure.

These results are consistent with previous observations about the number and nature of bonds and emphasize the importance of structural factors in studying CO_2_ adsorption processes on forsterite surfaces (Table 1).

To quantitatively evaluate the CO_2_ binding mechanisms on different forsterite surfaces, the adsorption energy was calculated. This value allows for determining the stability of the forming adsorption complexes and comparing the binding capacities of different surfaces. The adsorption energy was calculated using the following formula:

(1) $$E_{ads}=E_{{\mathrm{CO}}_{2}/FS}-E_{{\mathrm{CO}}_{2}}-E_{FS}$$

where $E_{{\mathrm{CO}}_{2}/FS}$ is the total energy of the system after adsorption, $E_{{\mathrm{CO}}_{2}}$ is the energy of the isolated CO_2_ molecule, and $E_{FS}$ is the energy of the clean forsterite surface. 

**Figure 2:**
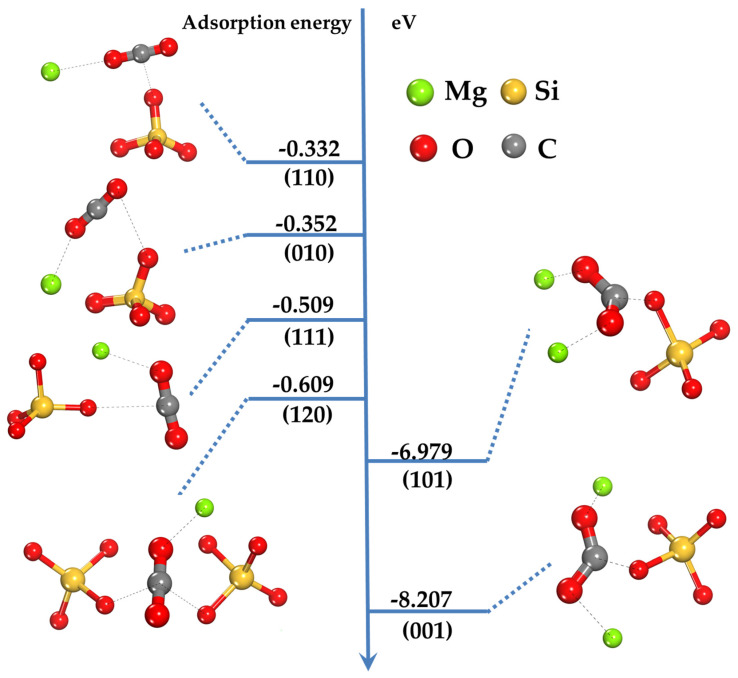
The adsorption energy (E_ads_) in eV of CO_2_ on forsterite surfaces.

Negative values of $E_{ads}$ indicate the exothermic nature of the adsorption process, demonstrating spontaneous interaction and energetically favorable formation of the adsorption complex (Figure 2). The more negative the adsorption energy, the stronger the interaction between CO_2_ and the surface, and the higher the stability of the forming complex.

The adsorption energies of CO_2_ on different forsterite surfaces are distributed in the following order: −8.21 eV for (001), −6.98 eV for (101), −0.61 eV for (120), −0.51 eV for (111), −0.35 eV for (010), and −0.33 eV for (110) (Figure 2). This corresponds to the sequence of surface adsorption capacity: (001) > (101) > (120) > (111) > (010) > (110). This order reflects the influence of Mg cations accessibility on the strength of interaction with CO_2_.

The obtained data show that CO_2_ adsorption energy significantly depends on the crystallographic orientation of the forsterite surface. The most negative the adsorption energy values are observed on the (001) and (101) surfaces, indicating stronger CO_2_ interaction with these surfaces compared to others.

The correlation between adsorption energy and the accessibility of Mg cations on the forsterite surface is clearly evident in the obtained results. The (001) and (101) surfaces are characterized by Mg cations available for interaction with CO_2_ molecules. This leads to the formation of strong chemical bonds between CO_2_ and the surface, which is confirmed by significant changes in molecule geometry after binding and high Mulliken bond populations (Table 1). On these surfaces, the CO_2_ molecule interacts with Mg cations through oxygen atoms, forming coordination bonds. This interaction leads to significant electron density redistribution and increased C–O bond lengths in the CO_2_ molecule, as well as a substantial decrease in the O–C–O bond angle, indicating molecule activation and strong interaction with the surface (Table 2).

On the (120), (111), (010), and (110) surfaces, Mg cations are less accessible or screened by oxygen layers, which limits direct interaction with CO_2_ molecules. Consequently, the adsorption energy on these surfaces is significantly less negative, indicating weaker interaction. In these cases, binding is predominantly driven by weak physical interactions, such as van der Waals forces, between the CO_2_ molecule and surface oxygen atoms. Changes in CO_2_ molecule geometry after binding on these surfaces are minimal: C–O bond lengths and O–C–O bond angle remain close to free molecule values. This indicates that the interaction does not lead to significant electron density redistribution in the CO_2_ molecule.

The obtained adsorption energy values correlate with the number and population of bonds between CO_2_ and the surface discussed earlier. The (001) and (101) surfaces with the highest number of bonds and high populations demonstrate the most negative adsorption energies. This confirms that strong chemical interaction and multiple bond formation leads to more stable adsorption complexes.

The strong adsorption energies observed on the (001) and (101) surfaces (−8.21 eV and −6.98 eV, respectively) suggest that these crystallographic orientations may be particularly important for subsequent mineralization processes. The formation of stable Mg–O bonds and significant electron density redistribution on these surfaces could potentially lower activation barriers for the conversion of adsorbed CO_2_ to carbonate species. However, the actual formation of stable magnesite (MgCO_3_) would require additional factors such as appropriate temperature, pressure, and the presence of water molecules to facilitate the necessary chemical transformations.

Conversely, surfaces with fewer bonds and lower populations show less negative adsorption energies, indicating weak interaction and less stable complexes.

These results align with previous studies, which also noted that the accessibility of metal cations on mineral surfaces significantly affects the adsorption energy of gaseous molecules [39,40,41]. Specifically, it was shown that Mg cations can actively interact with electronegative adsorbate atoms, enhancing binding through coordination bonds and electrostatic interaction.

Analysis of the CO_2_ binding mechanism on different forsterite surfaces showed that the strength and nature of interaction significantly depend on the crystallographic orientation of the surface and Mg cations accessibility. The most negative adsorption energies and significant changes in CO_2_ molecule geometry are observed on the (001) and (101) surfaces with accessible Mg cations, indicating strong chemical interaction. On surfaces with less accessible Mg cations, the interaction is weaker and predominantly driven by physical forces. These results emphasize the importance of structural and electronic factors in studying gas binding on minerals and may be valuable for improving CO_2_ mineralization processes, contributing to the development of more effective materials and methods for carbon dioxide capture and storage. 

### 2.2. Charge Analysis

The adsorption mechanism of CO_2_ molecules on different forsterite surfaces can be better understood by studying charge transfer between atoms in the forsterite-CO_2_ system. For this purpose, the electron density difference (Δρ) was calculated, which allows visualization of changes in electron density distribution during binding. The electron density difference was calculated using the formula:

$$\Delta \rho =\rho_{{\mathrm{CO}}_{2}/FS}-\rho_{{\mathrm{CO}}_{2}}-\rho_{FS}$$

where $\rho_{{\mathrm{CO}}_{2}/FS}$ is the electron density of the complete system after CO_2_ adsorption on the forsterite surface, $\rho_{{\mathrm{CO}}_{2}}$ is the electron density of the isolated CO_2_ molecule, and $\rho_{FS}$ is the electron density of the clean forsterite surface.

**Figure 3:**
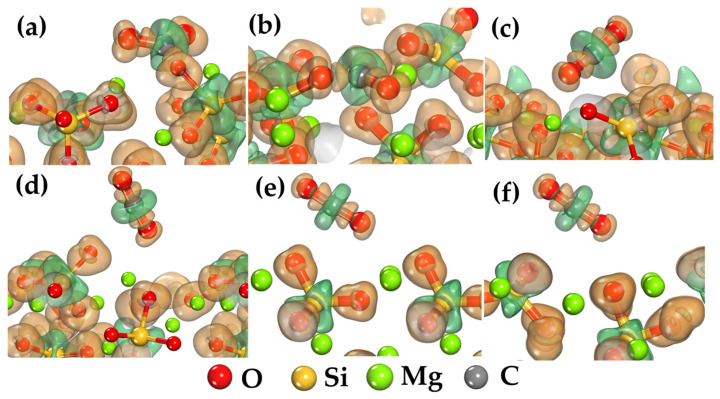
Electron density difference for CO_2_ adsorption on different forsterite surfaces: (**a**) (001), (**b**) (101), (**c**) (120), (**d**) (111), (**e**) (010), and (**f**) (110). The isosurface value is 0.1 electrons/Å³; orange regions indicate charge accumulation, while green regions show charge depletion.

As shown in Figure 3, the electron density difference corresponds to the Mulliken charges presented in Table 2. Green regions in the figure indicate charge depletion, while orange regions demonstrate charge accumulation. Of particular interest are the interaction regions between the CO_2_ molecule and the forsterite surface (Figure 3). The charge transfer and accumulation during CO_2_ binding occur through electron density depletion on the oxygen atoms of the CO_2_ molecule and its accumulation on surface magnesium cations.

The electron density difference analysis reveals distinct patterns of charge redistribution across different surface types. On surfaces with exposed Mg cations ((001) and (101)), the significant charge accumulation between CO_2_ oxygen atoms and surface Mg centers indicates the formation of strong ionic-covalent bonds. This charge redistribution is accompanied by substantial electron density depletion around the carbon atom of CO_2_, suggesting a complex bonding mechanism involving both charge transfer and orbital hybridization.

The surfaces dominated by oxygen termination show markedly different charge distribution patterns. On the (110) and (010) surfaces, the electron density redistribution is less pronounced and more diffuse, characteristic of weaker electrostatic interactions. The limited charge transfer and minimal localized charge accumulation regions support the predominance of physical binding mechanisms on these surfaces. This contrast in charge redistribution patterns helps explain the significant differences in adsorption energies and molecular deformation observed across different surface orientations.

On forsterite surfaces with accessible magnesium cations, such as (001) and (101), CO_2_ binding occurs primarily through interaction between the oxygen atom of the CO_2_ molecule and the surface Mg cation (Figure 1a,b). Analysis of the electron density difference shows significant electron density redistribution within the CO_2_ molecule.

An increase in negative charge on the oxygen atoms of CO_2_ is observed. On the (001) surface, the charge on oxygen atoms increases from −0.49e to −0.68e and −0.72e, while on the (101) surface, it increases to −0.75e and −0.74e. This indicates electron density accumulation on the oxygen atoms (Table 3). Additionally, there is a decrease in the positive charge on the carbon atom of CO_2_: on the (001) surface, the charge decreases from 0.98e to 0.66e, and on the (101) surface, to 0.72e. This indicates electron density loss from the carbon atom. This redistribution of electron density within the CO_2_ molecule may be attributed to the attraction of electron density from the carbon atom to the more electronegative oxygen atoms, enhancing molecular polarization during interaction with the surface (Table 3).

On forsterite surfaces (120), (111), (010), and (110), CO_2_ interaction with the surface occurs primarily through weak physical forces between CO_2_ carbon atoms and surface oxygen atoms (Figure 1c–f). Charge analysis shows less pronounced electron density redistribution. There is a smaller change in charges on CO_2_ oxygen atoms: charges change from −0.49e to approximately −0.40e to −0.44e. The positive charge on carbon shows minimal decrease: carbon charges are about 0.89e to 0.92e, which is close to the free molecule value (0.98e). This indicates that electron density redistribution within the CO_2_ molecule during binding on these surfaces is minimal, corresponding to weak van der Waals interaction character and absence of significant charge transfer between the molecule and surface (Table 3).

On the (001) and (101) surfaces, regions of electron density accumulation around CO_2_ oxygen atoms and depletion around carbon are visible, which is consistent with the charge data in Table 3. This indicates internal redistribution of electron density in the CO_2_ molecule during interaction with the surface (Figure 3a,b). On surfaces without accessible Mg cations, electron density changes are minimal, confirming weak interaction character and absence of significant charge transfer (Table 2, Figure 3c–f). The absence of surface Mg groups allows only weak interactions to form between CO_2_ carbon atoms and surface oxygen atoms. In this case, low-energy binding states occur without explicit electron density redistribution. During the formation of such weak interactions, charge transfer occurs to a minimal extent, which is consistent with the insignificant electron density changes shown in the figure.

### 2.3. Partial Density of States Analysis

To gain a deep understanding of the changes in electronic states of atoms involved in the binding of CO_2_ on various surfaces of forsterite, a detailed analysis of the partial density of electronic states (PDOS) was conducted. This method allows the investigation of changes in energy levels and the distribution of electron density on atomic orbitals during the interaction of a molecule with a surface, which is crucial for understanding the binding mechanism and the nature of the resulting bonds.

The analysis of PDOS shifts provides valuable insights into the nature and strength of the surface—CO_2_ interactions. The magnitude of the PDOS peak shifts correlates with the binding strength: larger shifts toward lower energies indicate stronger electronic interactions and more stable binding configurations. This correlation arises from the stabilization of electronic states when electrons occupy lower energy levels during bond formation.

**Figure 4:**
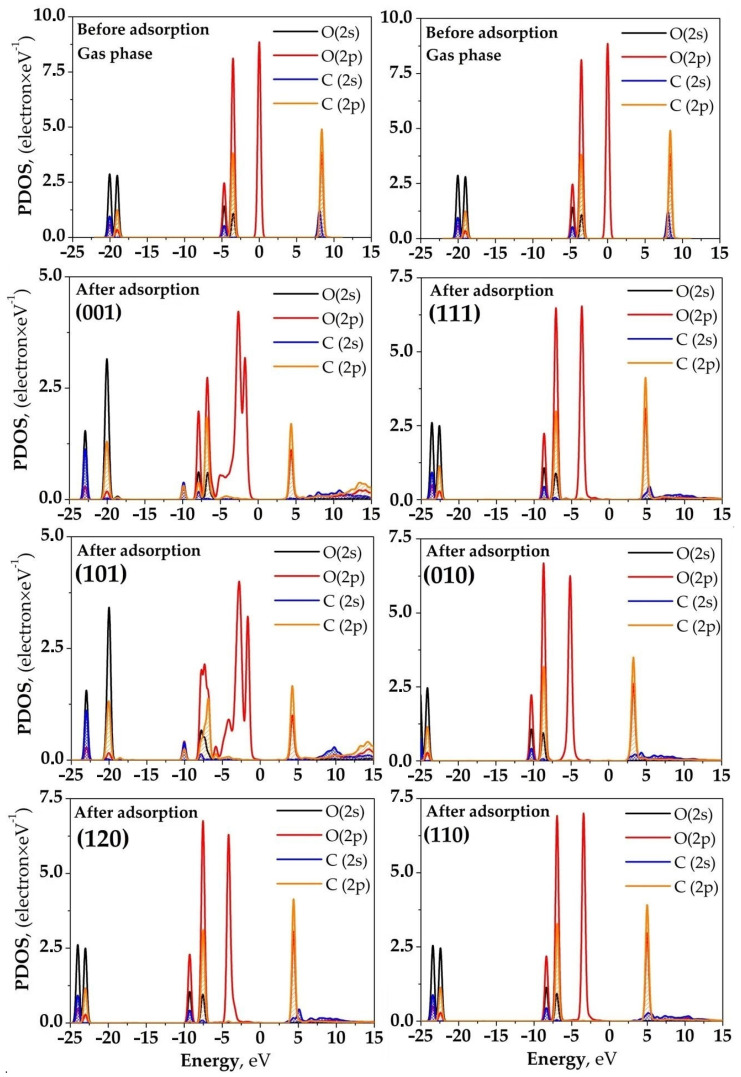
PDOS of the CO_2_ molecule before and after interacting with the surfaces of forsterite.

Figure 4 illustrates a comparison of the PDOS of the oxygen and carbon atoms of the CO_2_ molecule before and after binding on different forsterite surfaces, with the Fermi level set to 0 eV for ease of comparison. After the binding of the CO_2_ molecule on the forsterite surface, there is a shift of the PDOS peaks of the 2*p*-orbitals of the oxygen atoms of CO_2_ toward lower-energy (deeper) levels. This indicates a decrease in the energy of the valence electrons of oxygen, which may be associated with the formation of chemical bonds and the redistribution of electron density. Simultaneously with the energy shift, a reduction in the peak heights of the density of states is observed. This suggests a decrease in the availability of electronic states for electron transfer, which may lead to a reduction in the reactivity of the CO_2_ molecule after binding.

The most significant changes in the partial density of states (PDOS) are observed on the (001) surface of forsterite. The PDOS peaks of the 2*p*-orbitals of the oxygen atoms in CO_2_ shift by 1.7 eV towards lower energies compared to those of the isolated CO_2_ molecule. This substantial shift indicates a strong interaction between the molecule and the surface. The maximum density of states of the 2*p*-orbitals of oxygen in CO_2_ decreases from 8.8 to 3.2 electrons/eV. Such a reduction in peak intensity may be associated with the partial filling of these states during the formation of chemical bonds with the surface. 

The significant downward shift of 1.7 eV in the PDOS peaks of oxygen 2*p*-orbitals indicates strong orbital hybridization between CO_2_ and surface atoms. This hybridization results in the formation of new bonding and antibonding molecular orbitals, observed as redistributed peaks in the PDOS spectrum. The strong orbital overlap leads to electron localization in the bonding regions, contributing to the high stability of the adsorption complex on the (001) surface.

**Figure 5:**
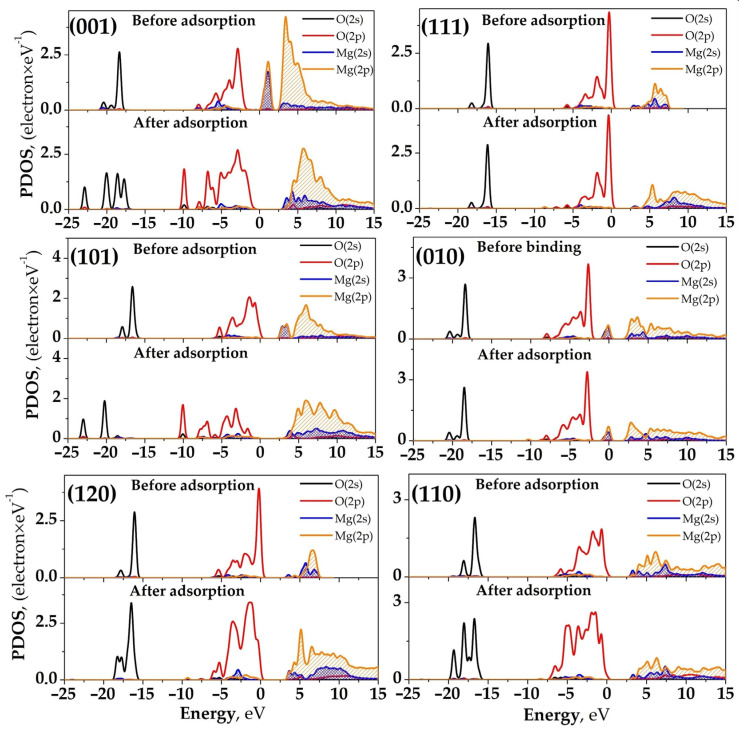
PDOS of the surface atoms of forsterite before and after CO_2_ adsorption.

Particularly notable changes in electronic structure are observed for the (001) and (101) surfaces, where CO_2_ binding leads to a significant increase in the band gap width. As shown in Figure 5, this electronic transformation is evidenced by a substantial decrease in the density of states near 0 eV, indicating reduced reactivity of these surfaces after CO_2_ binding. The changes are particularly pronounced for these surfaces due to their higher adsorption energies and stronger interaction with CO_2_ molecules. Other forsterite surfaces show less significant changes in their electronic structure near the Fermi level, which correlates with their lower adsorption energies and consequently weaker surface-CO_2_ interactions.

The observed band gap expansion on these surfaces is accompanied by significant orbital hybridization between the 2*p*-orbitals of CO_2_ oxygen atoms and the surface O atoms’ 2*p* orbitals. The emergence of new peaks in the PDOS spectrum indicates the formation of hybrid molecular orbitals, while the reduction in the density of states near the Fermi level suggests electron localization in the newly formed bonds. Changes in the shape and distribution of PDOS peaks reflect modified electronic configurations. These hybridization effects enhance the covalent character of the surface-adsorbate interaction and contribute to the overall stability of the adsorption complex.

On other forsterite surfaces, such as (101) and (120), similar trends in PDOS changes are observed after CO_2_ binding, although the magnitude of these changes may be less pronounced. The shifts in energy levels and the decrease in the density of states of the 2*p*-orbitals of the oxygen atoms in CO_2_ after binding indicate a redistribution of electron density within the system and alterations in the electronic properties of the CO_2_ molecule. The lowering of the valence electron energy of oxygen suggests stabilization of the electronic states of the oxygen atoms due to interaction with the surface. This stabilization is particularly evident in the band gap width expansion for (001) and (101) surfaces after CO_2_ binding, which can be attributed to the strong interaction between the adsorbate and surface. These electronic modifications may also be associated with an increased ionic character of the bond between the oxygen of CO_2_ and the surface Mg cation (Figure 5). The reduction in the reactivity of the CO_2_ molecule, caused by the decreased density of states near the Fermi level, can lead to reduced availability of electrons for chemical reactions. This indicates strong binding of the molecule to the surface, resulting from the hybridization of the 2*p*-orbitals of oxygen in CO_2_ with the Mg orbitals, leading to the formation of new molecular orbitals distributed between the molecule and the surface.

The analysis of the partial density of electronic states (PDOS) confirmed that the binding of CO_2_ on forsterite surfaces leads to significant changes in the electronic states of both the molecule and the surface. The observed changes in electronic structure, particularly the band gap width expansion on (001) and (101) surfaces, demonstrate how CO_2_ binding can fundamentally alter the electronic characteristics of the surface, affecting its chemical reactivity and surface properties. On surfaces with accessible Mg cations, such as (001) and (101), significant shifts in energy levels and a decrease in the density of states are observed, indicating strong interaction and the formation of new chemical bonds. This results in the stabilization of the adsorption complex and corresponds to high adsorption energies on these surfaces.

On surfaces with less accessible Mg cations, the PDOS changes are less pronounced, indicating weaker interaction and the predominance of physical forces in the binding mechanism. These results underscore the importance of electronic effects in adsorption processes and may be beneficial in the development of new materials for the effective capture and storage of CO_2_.

## 3. Materials and Methods

### 3.1. Models

**Figure 6:**
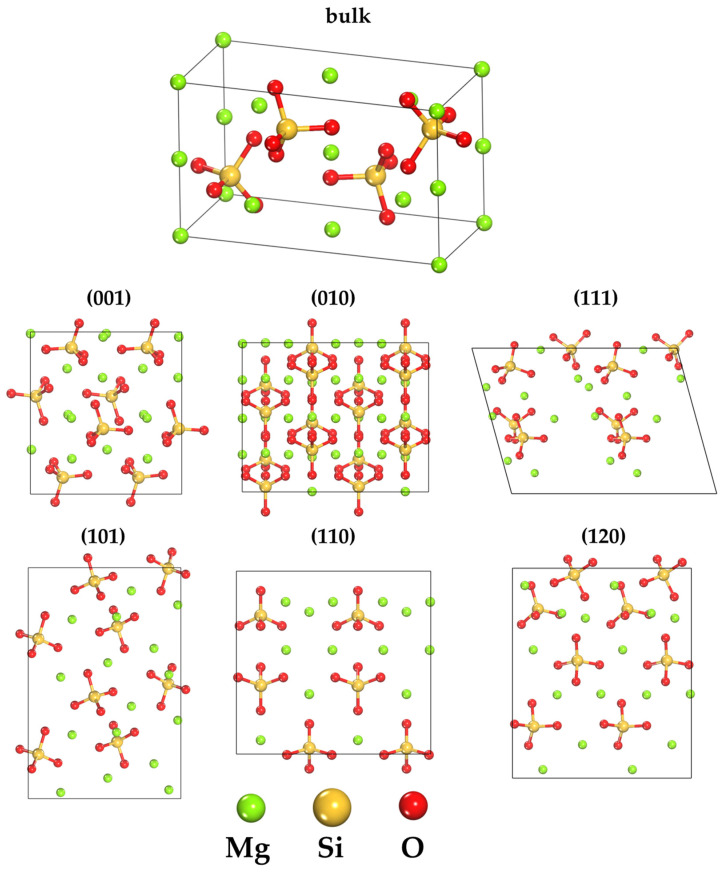
Forsterite bulk and side views of the six slab models: atomically smooth (001) surface dominated by exposed Mg atoms; ideal (010) surface terminated by SiO_4_ tetrahedra with coordinatively unsaturated Mg sites; high-index (111) surface featuring multiple distinct binding sites; reconstructed (110) surface with modified surface Mg coordination; reconstructed (120) surface showing exposed octahedral Mg centers.

The initial model of forsterite was used with the following unit cell parameters: a = 4.756 Å, b = 10.207 Å, c = 5.980 Å and angles: α = β = γ = 90°, which corresponds to orthorhombic syngony with space group Pbnm (Z = 4) [42]. Forsterite (Mg_2_SiO_4_) is the magnesium last member of the isomorphic series of olivines (Mg,Fe)_2_SiO_4_. Its crystal structure consists of a distorted hexagonally densely packed oxygen lattice, where 1/8 of the tetrahedral positions is occupied by silicon ions Si⁴⁺, forming SiO_4_ groups, and 1/2 of the octahedral positions is occupied by divalent magnesium ions Mg^2^⁺, forming MgO₆ units (Figure 6).

The surfaces selected for this study—(010), (101), (001), (110), (111), and (120)—were generated by sectioning the forsterite crystal along specific crystallographic planes (Figure 6). The adsorption surfaces contain either Mg^2^⁺ cations or oxygen atoms of the outer SiO_4_ groups. These surfaces were chosen for their prevalence and stability in natural settings, as well as for their structural and morphological diversity, which represents the primary low-index planes of forsterite commonly observed in geochemical processes. The stability of these planes, confirmed by prior research, suggests they are significant for geochemical interactions and adsorption processes [31,35].

The six surfaces under consideration are characterized by a variety of structural features and morphologies. Some of them, such as surfaces (010) and (001), have a compact structure with a small number of available sites and a small surface area. In contrast, surfaces (120), (101) and (111) have more complex morphologies, providing many sites for binding. The (110) surface is characterized by the presence of low-coordinated Mg^2^⁺ sites, which explains its high surface energy [35].

It is important to note that defects, such as steps, corners, and vacancies, were not considered in the surface models. The presence of such defects does not significantly affect the energetic parameters of the adsorption process; a more significant factor is the presence of open and low-coordinated Mg^2^⁺ cations [31].

The CO_2_ molecule was optimized in a cubic cell with dimensions 30 Å × 30 Å × 30 Å and a Gamma point in k-space. The CO_2_ optimization was carried out with the same parameters as for the forsterite surface models.

### 3.2. Methods

CASTEP software version 7.01 (CDG,UK) was used for DFT calculations [43]. Exchange-correlation interactions were described in the framework of the generalized gradient approximation (GGA) using the Perdew–Burke–Ernzerhof (PBE) functional theory [44]. The choice of the PBE functional was motivated by its widespread use and proven reliability in modeling adsorption processes on oxide surfaces, including silicate minerals. The PBE functional offers a good balance between accuracy and computational efficiency for systems of this complexity and size [45]. To account for dispersion interactions, the Grimme method in the DFT-D2 approximation [46] was used, which allows the van der Waals forces in the system to be correctly accounted for. Incorporating the DFT-D2 dispersion correction is essential for accurately capturing van der Waals interactions, which play a significant role in the physisorption of small molecules [47].

The geometry of the models was optimized using the BFGS algorithm and the following convergence criteria: maximum atomic displacement was 5 × 10^−4^ Å, maximum interatomic force was 0.01 eV/Å, maximum pressure was 0.02 GPa. The convergence of the self-consistent field was achieved for energy changes less than 5 × 10^−7^ eV/atom [48]. During the geometric optimization, all atoms in the slab models were fully relaxed without any constraints. No atoms were fixed during the optimization process. This approach ensures that both surface and subsurface atomic positions can adjust freely to achieve the lowest-energy configuration, capturing any relaxation or reconstruction effects throughout the slab.

The cutoff energy of the plane wave basis was set at 700 eV, which provides an optimal balance between the calculation accuracy and computational cost. Ultra-soft Vanderbilt pseudopotentials were used to describe the interaction of valence electrons with the ionic backbone [49]. The following electronic configurations were chosen as valence states: Mg(2p^6^3s^2^), Si(3s^2^3p^2^), O(2s^2^2p^4^), and C(2s^2^2p^2^), which provides an accurate reproduction of the electronic structure of materials.

The Brillouin zone integration was performed using k-points according to the Monkhorst–Pack scheme: a 4 × 2 × 3 grid was used for the forsterite unit cell, and a 2 × 2 × 1 grid was used for the surface models [50]. This choice provides sufficient accuracy at an acceptable computational cost.

## 4. Conclusions

In this study, the mechanism of CO_2_ adsorption on various surfaces of forsterite was investigated using density functional theory (DFT) methods. It was established that the CO_2_ molecule can stably bind on forsterite surfaces, and the binding occurs due to interactions between the oxygen atoms of CO_2_ and the surface magnesium and oxygen atoms of the mineral.

The binding mechanism is governed by electrostatic interactions and partial charge transfer from the CO_2_ molecule to the forsterite surface. Analysis of the electron density difference and the partial density of electronic states (PDOS) showed that after binding, the energy levels of the oxygen atoms in CO_2_ shift to lower energies, and there is a decrease in the peaks of the density of states. This indicates a reduction in the reactivity of the CO_2_ molecule and the formation of weak covalent interactions with the mineral surface.

The adsorption energy of CO_2_ varies widely, from −0.33 eV for the (110) surface to −8.21 eV for the (001) surface. Such a range of values indicates a significant influence of the crystallographic orientation of the forsterite surface on its binding mechanism. The (001) and (101) surfaces demonstrate the strongest interaction with the CO_2_ molecule. This is confirmed by the most negative adsorption energy values and significant changes in the geometry and electronic structure of the molecule after adsorption.

The obtained results deepen the understanding of CO_2_ binding mechanisms on forsterite surfaces and emphasize the importance of the mineral’s crystallographic orientation and electronic properties. A key role in adsorption is played by the availability of Mg cations, which are capable of forming partially covalent bonds with the CO_2_ molecule, significantly increasing the interaction energy and stability of the resulting complexes.

This study provides a theoretical basis for understanding the initial steps of CO_2_ capture on forsterite surfaces. While our focus has been on the fundamental binding mechanisms, the observed strong interactions and electronic structure modifications, particularly on surfaces with accessible Mg cations, may have important implications for subsequent mineralization processes. The formation of stable magnesite (MgCO₃) would require additional environmental factors beyond simple adsorption, including appropriate temperature, pressure, and the presence of water. Future studies combining our understanding of surface-specific binding mechanisms with these environmental parameters could help optimize conditions for enhanced CO_2_ mineralization rates in both natural and engineered systems.

## Figures and Tables

**Table 1:** The structural parameters of the likely bonds on the low-energy adsorption configurations.

Bonding Type	Nb ^†^	001	101	120	111	010	110
Mg–O	1	2.053 (0.89)	1.976 (1.25)	2.703 (0.10)	2.219 (0.54)	2.343 (0.25)	2.870 (0.01)
	2	1.999 (1.03)	1.971 (1.28)	2.261 (0.43)	-	-	-
Mg–C	3	2.548 (0.58)	2.901(0.26)	-	2.982 (0.28)	2.745 (0.00)	-
	4	2.888 (0.24)	-	-	-	-	-
O–C	5	1.389 (0.67)	1.360 (0.67)	2.963 (0.00)	2.749 (0.01)	-	2.949 (0.00)
	6	-	-	2.835 (0.10)	-		-
O–O	7	2.205 (0.34)	2.244 (0.19)	-	2.827 (0.07)		2.923 (0.03)
	8	2.300 (0.18)	2.245 (0.20)	-	-	-	-
	9	2.930 (0.04)	-	-	-	-	-

^†^ from Figure 1, Nb—number of bonds.

**Table 2:** The structural parameters of CO_2_ in the lowest-energy adsorption configurations.

Forsterite Surfaces	O_c_–C (Å)	C–O_c_ (Å)	∠O_c_–C–O_c_ °
Free CO_2_	1.180	1.180	179.753
001	1.267	1.269	128.058
101	1.268	1.270	125.468
120	1.168	1.192	175.546
111	1.193	1.169	175.899
010	1.187	1.173	176.096
110	1.186	1.174	177.436

**Table 3:** Values of the charges on the atoms of the CO_2_ molecule after adsorption on various forsterite surfaces.

Forsterite Surfaces	O_c_ (e)	C (e)	O_c_ (e)
Free CO_2_	−0.49	0.98	−0.49
001	−0.68	0.66	−0.72
101	−0.75	0.72	−0.74
120	−0.40	0.89	−0.57
111	−0.42	0.92	−0.56
010	−0.42	0.91	−0.52
110	−0.44	0.92	−0.52

## Data Availability

The data presented in this study are available upon request from the corresponding author.
